# In Vitro Gene Expression Responses of Bovine Rumen Epithelial Cells to Different pH Stresses

**DOI:** 10.3390/ani12192621

**Published:** 2022-09-29

**Authors:** Hongxia Lian, Chuankai Zhang, Yifan Liu, Wenjing Li, Tong Fu, Tengyun Gao, Liyang Zhang

**Affiliations:** 1 College of Animal Science and Technology, Henan Agricultural University, Zhengzhou 450046, China; 2 Henan International Joint Laboratory of Nutrition Regulation and Ecological Raising of Domestic Animal, Zhengzhou 450046, China

**Keywords:** apoptosis, dairy cows, inflammation, metabolomics, pH, RNA-Seq

## Abstract

**Simple Summary:**

Dietary changes and improper pasture feeding management can lead to subacute rumen acidosis or acute rumen acidosis in cattle. When acidosis occurs, the rumen pH rapidly decreases to an abnormal level and induces lipopolysaccharide (LPS) release by rumen microorganisms. The entry of LPS into the blood through the rumen epithelium induces systemic inflammatory reactions that ultimately result in body damage, reduced performance, and even the death of animals. However, the manner in which genes in the rumen epithelium are regulated in response to pH reduction remains unclear. In this study, transcriptome sequencing and metabolome analysis were performed on rumen epithelial cells at different pH values in vitro to determine the adaptive expressions of genes and metabolites in rumen epithelial cells during the occurrence of rumen acidosis. Our results contribute to a deeper understanding of the response mechanism of the rumen epithelium during the occurrence of rumen acidosis.

**Abstract:**

Ruminal acidosis often occurs in production, which greatly affects animal health and production efficiency. Subacute rumen acidosis (SARA) occurs when rumen pH drops rapidly to 5.5–5.8, and acute rumen acidosis (ARA) occurs when rumen pH drops below 5.0, but the molecular regulation mechanism of the rumen epithelium after the rapid decrease in pH is still unclear. Bovine rumen epithelial cells (BRECs) were cultured at pH = 7.4 (control), 5.5 (SARA), and 4.5 (ARA). Transcriptome and metabolomic methods were used to obtain the molecular-based response of BRECs to different pH treatments; pH = 4.5 can significantly induce apoptosis of BRECs. The RNA-seq experiments revealed 1381 differently expressed genes (DEGs) in the control vs. SARA groups (*p* < 0.05). Fibroblast growth factor (FGF) and tumor necrosis factor (TNF) were upregulated 4.25 and 6.86 fold, respectively, and TLR4 was downregulated 0.58 fold. In addition, 283 DEGs were identified in the control vs. ARA comparison (*p* < 0.05), and prostaglandin-endoperoxide synthase 2 (PSTG2) was downregulated 0.54 fold. Our research reveals that the MAPK/TNF signaling pathway regulates the inflammatory response of BRECs. Metabolomics identified 35 biochemical compounds that were significantly affected (*p* < 0.05) in control vs. SARA and 51 in control vs. ARA. Bioinformatics analysis using the Kyoto Encyclopedia of Genes and Genomes (KEGG) pathway database revealed that drug metabolism-cytochrome P450 metabolic and alpha-linolenic acid metabolism changes occurred. These transcriptional and metabolic changes are related to the adaptation of BRECs to low-pH stresses. In conclusion, the combined data analyses presented a worthy strategy to characterize the cellular, transcriptomic, and metabonomic adaptation of BRECs to pH in vitro. We demonstrated transcriptional expression changes in BRECs under pH stress and activation of the molecular mechanisms controlling inflammation.

## 1. Introduction

Both metabolizable energy and dry matter intake (DMI) must be adjusted to maximize milk production [1]. Therefore, the diet of high-yielding cows is supplemented with grain or particulate matter [2]. The ruminal environment may drastically shift in response to the abrupt addition or increase of starch during feeding [3]; the composition and metabolism of the ruminal microbiota changes, which causes a buildup of volatile fatty acids (VFAs) and a sharp decline in rumen pH [4,5]. Therefore, rumen acidosis is common in dairy cows. Depending on the extent of the clinical manifestations of rumen acidosis, the end result may be subacute ruminal acidosis (SARA) or acute ruminal acidosis (ARA). When ruminal acidosis occurs, the rumen is continuously exposed to pH < 5.0. Subacute ruminal acidosis has no clinical manifestations and is defined as rumen pH dropping from 5.5 to 5.0 and remaining in that range for 111 to 180 min over a 24 h period [6]. According to research in the dairy industry, approximately 20% of high-producing dairy cows have SARA, which has raised significant concerns for the industry [7]. In dairy cows, SARA has been proven to impact feed intake, milk production, rumen microflora, and rumen digestion, and can lead to diarrhea, rumen mucosal injury, laminitis, rumenitis, and other conditions. The ARA condition develops more rapidly and more seriously [8,9,10].

Previous studies have shown that grain-induced SARA raises the levels of acute-phase proteins in peripheral blood, including haptoglobin and serum amyloid A, which signify a systemic inflammatory response [11]. A high-grain diet disrupts the function of the rumen epithelium, including cell necrosis and disruption of tight junctions [12]. This promotes the migration of microorganisms and immunogenic substances into the portal circulation [13], causing further systemic inflammation. Lipopolysaccharide (LPS) and lowered pH may operate synergistically to alter epithelial barrier function and ultimately breach the epithelial barrier, according to a study that employed isolated rumen and colon tissues from steers [14]. After fracture of the epithelium, LPS and endotoxin are free to translocate through the damaged ruminal wall [15,16]. The mRNA expression levels of IL-1β, IL-2, IL-6, and IL-8 increased during the interaction of LPS with BRECs, suggesting that these may be mainly responsible for the triggered ruminal inflammation [17]. Nevertheless, systematic research on the mechanism of pH reduction on BRECs is rare.

Therefore, the objective of our study was to simulate rumen acidosis with butyric acid in vitro and investigate the mechanism of its effects by transcriptomics and metabolomics. This study expands our knowledge regarding the molecular mechanism of BREC inflammation under low-pH conditions and identifies molecular pathways that are significantly regulated in acidotic BRECs, which could be used to attenuate the detrimental impact of ruminal acidosis and provide a theoretical basis for feeding high-yield dairy cows with high concentrate.

## 2. Materials and Methods

### 2.1. Cell Isolation and Culture

All procedures involving animals were approved by the Institutional Animal Care and Use Committee (IACUC) of Henan Agriculture University (Permit Number: 12-1328; Date: 05-2021). Rumen tissue was collected from a 3-day-old calf that was stunned by electric shock and killed by exsanguination. BRECs were isolated following the protocol of Klotz [18] with slight modifications. Upon collection, the rumen tissue was first rinsed with ice-cold normal saline, then with phosphate-buffered saline (PBS), and then with PBS supplemented with 5% antibiotic–antimycotic (ABAM) (100×). The minced tissue was digested in 0.25% trypsin-EDTA (Gibco, Grand Island, NY, USA) for 30 min at 37 °C. We repeated the above digestion steps several times and observed the tissue in real time with an optical microscope. After digestion, the tissue was filtered through a 300-µm nylon mesh. Centrifuging the filtrate at 100× *g* for 10 min allowed the cells to be recovered. The cells were then resuspended in growth medium composed of Dulbecco’s modified Eagle’s medium (DMEM/F-12) (Gibco) and 10% fetal bovine serum (FBS, Gibco) after being washed three times with PBS supplemented with 2% ABAM. Before being frozen in liquid nitrogen for preservation, the cells were grown for three days at 37 °C in a humidified incubator with 5% CO_2_. Except where otherwise noted, all cell culture media and reagents were purchased from Biological Industries (Kibbutz Beit-Haemek, Israel).

### 2.2. Immunofluorescence Identification of BRECs

To identify the origin of the primary cell culture, immunostaining was performed. After confluence, cell cultures in 8-well plates at a density of 1 × 10^5^ cells per well were fixed with 4% paraformaldehyde (Solarbio, Beijing, China) at 4 °C for 30 min. The cells were then permeabilized with 0.3% Triton X-100 (Sigma-Aldrich, St. Louis, MO, USA) for 20 min. Then, the cells were blocked with 10% FBS (Gibco) and 1%BSA (Solarbio) in PBS. The cells were incubated overnight with primary mouse anti-cytokeratin 18 antibody (1:200: ab668, Abcam, Boston, MA, USA) at 4 °C and then incubated for 2 h with secondary fluorescein isothiocyanate (FITC)-labeled goat anti-mouse IgG antibodies (1:200, PeproTech, East Windsor, NJ, USA) at room temperature in the dark. The cell nuclei were stained with 4′,6-diamidino-2-phenylindole (DAPI, Solarbio, Beijing, China) prior to adding an anti-fluorescence quenching agent and covering the slides with coverslips. A laser scanning confocal microscope was used to investigate fluorescent staining (NikonEclipse TS100, Nikon Corporation, Tokyo, Japan).

### 2.3. Cell pH Treatment

BREC cells (1 × 10^6^ cells per well) were precultured in a 6-well plate. Cells were more than 80% confluent and adhered to the bottom of the wells after 48 h of preculture. BRECs were then cultured at different pH values, namely, control group (pH = 7.4), SARA group (pH = 5.5), and ARA group (pH = 4.5), for 6 h [19,20]. The pH levels of the three groups were adjusted by adding butyrate (0, 10, 20 mmol/L, Sigma, St. Louis, MO, USA) to the DMEM/F-12 media. After cultivation, the cells and culture media were collected for additional examinations.

### 2.4. Estimation of Cell Viability and Apoptosis

Cell viabilities were measured using the Cell Counting Kit-8 (Meilun, China) according to the manufacturer’s instructions. Cells (1 × 10^4^ cells/mL) were plated in 96-well plates at 37 °C for 4 h. Cells were treated with butyrate at pH = 7.4, 5.5, or 4.5 for 6 h. After treatment, the cells were incubated with 10 µL of Cell Counting Kit-8 solution at 37 °C for 2 h. The absorbances were measured at 450 nm with a microplate reader (ThermoFisher Scientific, Grand Island, NY, USA). Cell viabilities were calculated as percentages relative to the control cells.

After grouping treatment, apoptosis was detected using an annexin V-FITC apoptosis detection kit (US Everbright Inc., Suzhou, China). The adherent cells treated in each group were washed with PBS, digested with trypsin, and centrifuged (300× *g*, 5 min, 4 °C) to collect the cells. We added V-FITC to each group of samples according to the instructions of the kit and analyzed them by flow cytometry (Becton Dickinson, San Jose, CA, USA).

### 2.5. RNA-Seq and Data Analysis

Library preparation, construction, and sequencing were performed by Shanghai Majorbio Bio-Pharm Biotechnology Co., Ltd. (Shanghai, China). Briefly, the library preparations used the TruSeq^TM^ RNA Sample Preparation kit from Illumina (San Diego, CA, USA). The library was sequenced using the Illumina HiSeq xten/NovaSeq 6000 sequencer, and 2 150-bp paired-end reads were produced after the library quality had been evaluated by TBS380 (Turner Bio-Systems, Sunnyvale, CA, USA). With default settings, SeqPrep (https://github.com/jstjohn/SeqPrep (accessed on 22 February 2021)) and Sickle (https://github.com/najoshi/sickle (accessed on 22 February 2021)) were used to trim and quality-check the raw paired-end reads [21,22]. The reference genome was then used as a reference for clean read alignment using TopHat software (version 2.0.0, available at http://tophat.cbcb.umd.edu (accessed on 25 February 2021)) [23].

### 2.6. Transcriptome Analysis

The expression level of each transcript was calculated using the metric ‘fragments per kilobase of transcript per million mapped reads’ (FPKM) in order to identify the differentially expressed genes (DEGs) [24]. Gene abundances were measured using RSEM (http://deweylab.biostat.wisc.edu/rsem/ (accessed on 9 March 2021)) [25]. DESeq2 (a method for differential analysis of count data in R, http://www.bioconductor.org/packages/release/bioc/html/DESeq2.htm (accessed on 9 March 2021)), a statistical package for R, was used to identify the DEGs [26]. A *p* < 0.05 and [log2 (fold change)] > 1 were used to determine the DEGs. Goatools was used to analyze the gene ontology (GO) enrichments of the DEGs [27]. The statistical enrichments of DEGs in the KEGG pathways were examined using the R programming language [28].

### 2.7. Construction of the Protein-Protein Interaction (PPI) Network

String (https://string-db.org/ (accessed on 24 April 2022)) and NetworkX (https://networkx.org/ (accessed on 24 April 2022)) were used to create the PPI network of DEGs enriched in signaling pathways [29,30].

### 2.8. Quantitative Reverse Transcription PCR (RT-qPCR) Validation for RNA-Seq

To validate the sequencing data, RT-qPCR analysis was performed using ChamQ SYBR qPCR Master Mix (Vazyme Biotec, Nanjing, China) on a Light Cycler 480 system (Roche, Basel, Switzerland). Six DEGs were randomly selected for determinations and primer blasts (http://www.ncbi.nlm.nih.gov/tools/primer-blast/ (accessed on 22 March 2021)) were used to design gene-specific primers (Supplementary Materials Table S1). The gene expression levels were normalized to the expression of GAPDH and were calculated by the 2^−ΔΔCt^ method [31]. All samples were tested in three technical replicates on the same plate.

### 2.9. Metabolomics

To investigate the pH-induced metabolic changes in BRECs, cell culture supernatants were subjected to LC–MS analyses (*n* = 6 in each experimental group). The supernatant was stored at −80°C prior to the ultra-performance liquid chromatography with mass spectrometry (UPLCMS; ThermoFisher, Waltham, MA, USA) experiments. The samples (2 μL) were detected using an ultra-performance liquid chromatography coupled with a mass spectrometry system (Q Exactive HF-X, Thermo Fisher, Waltham, MA, USA) in full-scan monitoring mode from m/z 70 to m/z 1050.

Progenesis QI software (Waters Corporation, Milford, CT, USA) and the free online Majorbio Cloud Platform were used to evaluate the raw data [32]. Metabolites were discovered based on the (Metabolite Link) METLIN database and Human Metabolome Database (HMDB) [33,34]. Equal volumes of extracted samples were combined to create quality control samples, which were then examined using the same procedure as the analytical samples. Data with relative standard deviations (RSDs) greater than 30% were discarded prior to the metabolomic analysis utilizing quality control (QC) verification. Principal component analysis (PCA) and partial least squares-discriminate analysis (PLS-DA) were carried out using R software (Version 1.6.2). The threshold of the variable importance in projection value (VIP) was established at 1 using the orthogonal partial least squares discriminant analysis (OPLS-DA) model for differentially choosing metabolites among various treatment groups. The Stats package in R and the SciPy package in PYTHON were used to analysis the metabolic pathways and metabolite set enrichments [35].

### 2.10. Integrated Transcriptome and Metabolome Analysis

Pearson correlation tests were applied to the gene expression and metabolite content data to find correlations among discriminant gene expressions and discriminant metabolite contents. Only associations that had *p* values of 0.05 or greater were selected.

### 2.11. Statistical Analysis

Data are expressed as the mean values ± SEMs. SPSS 16.0 was used for the statistical analysis. One-way ANOVA and Duncan’s test were used for post hoc tests, and the differences in cell apoptosis, cell viability, and RT-qPCR results between the control and treatment groups were examined. Differences were considered to be statistically significant at *p* < 0.05.

## 3. Results

### 3.1. Isolation and Identification of Keratin 18 in BRECs

**Figure 1:**
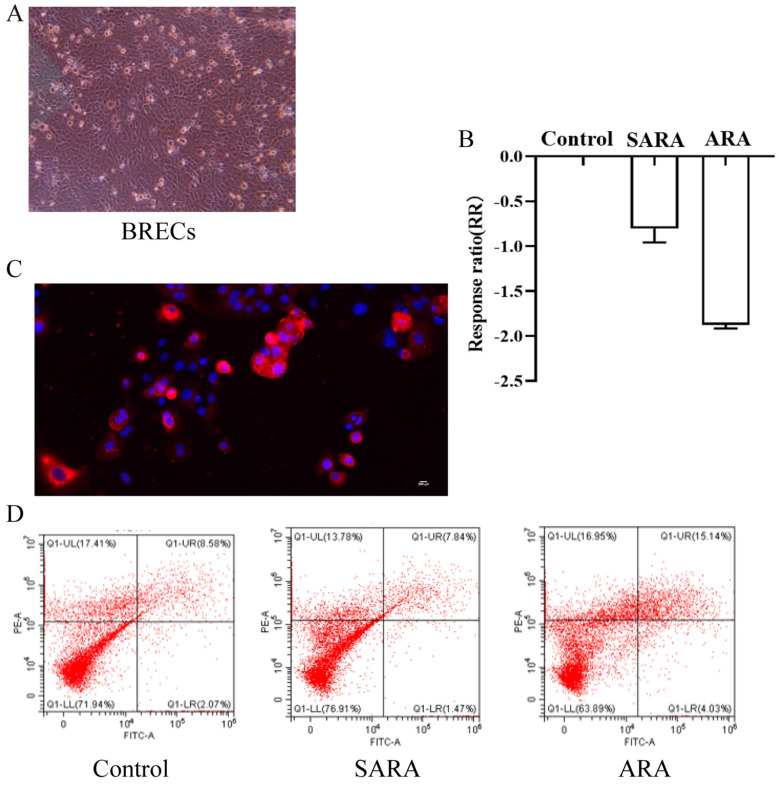
Effects of the morphology and pH of BRECs on their viability and apoptosis. (**A**). Morphology of BRECs (100×) (**B**). Effect of pH treatments on the viability of rumen epithelial cells. The abscissa represents the processing group, ordinate RR = ln (ODtreated/ODuntreated), optical density (OD); ODtreated indicates the OD values of treated cells, ODuntreated indicates the OD values of cells without treatment, RR > 0 indicates an increase in the relative viability of cells treated with butyrate compared with untreated cells, and RR < 0 indicates a decrease in the relative viability of cells treated with butyrate compared with untreated cells. (**C**). Immunofluorescence staining of keratin 18 in BRECs. Red indicates keratin 18 and blue indicates the nucleus. (**D**). Apoptotic rates of BRECs detected by flow cytometry. PE = phycoerythrin; FITC = fluorescein isothiocyanate.

Cells were treated with 1 × 10^6^/mL deposited into a 25 cm^2^ culture flask, purified, and cultured for three days (Figure 1A). To verify that the isolated cells originated from the epithelial cell type, the cultured cells were identified by immunofluorescence. The results showed that the isolated cells could express the epithelial skeleton protein, keratin 18; that is, the BRECs could emit red fluorescence when keratin 18 secondary antibody was added (Figure 1B).

### 3.2. Effect of pH on BREC Viability and Apoptosis

The effects of pH treatments on the viability of BRECs in vitro are shown in Figure 1C. The results showed that the different pH treatments significantly reduced the viability of BRECs (*p* < 0.01). The BREC apoptotic rates were estimated by flow cytometry (FCM) and fluorescence microscopy (Figure 1D). Compared with the SARA group, the ARA group had a significantly reduced proportion of normal cells (*p* < 0.05). The pH treatment did not affect the proportions of necrotic cells and early apoptotic cells (*p* > 0.05). Compared with the control and SARA groups, the ARA group had a significantly increased proportion of late apoptotic cells and scores on the apoptosis index (*p* < 0.05) (Table 1).

### 3.3. Transcriptomic Data and Detection of Expressed Genes in Samples

**Figure 2:**
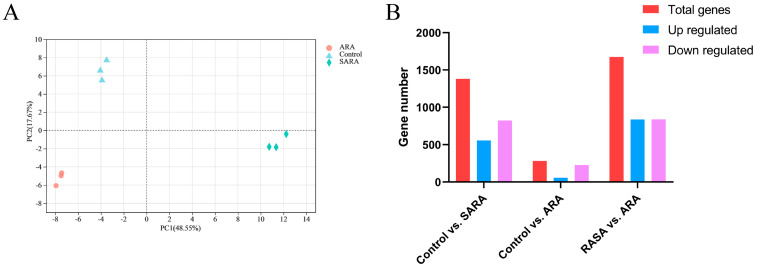
PCA of gene expressions and numbers of DEGs in the BRECs. (**A**). PCA of gene expressions in the three groups of BRECs. (**B**). Graphical representation of significant DEGs detected “control vs. SARA”, “control vs. ARA”, and “SARA vs. ARA” in BRECs cultured at different intensities at different pH values.

In order to investigate the variations in mRNA transcriptional expression between the three groups, RNA-Seq was used to identify the differentially expressed genes (DEGs) of BRECs. Nine BREC samples produced a total of 481.72 million (53.52 ± 5.37 million reads per sample) high-quality, paired reads, and the overall read alignment rate to the cattle reference genome was 93.78% ± 0.23%. Total gene expressions from the BREC samples’ principal component analysis (PCA) clearly clustered the three groups. (Figure 2A). The filter criteria for significant differential expressions were an adjusted *p* < 0.05 and absolute value of [log2 (FC)] > 1, which were used to count the differentially expressed genes (DEGs). A total of 1381 DEGs, including 558 upregulated and 823 downregulated DEGs, were identified in control vs. SARA. Similarly, for control vs. ARA, a total of 283 DEGs with 56 up- and 227 downregulated DEGs were reported. For SARA vs. ARA, 1674 significant DEGs were detected, including 836 up- and 838 downregulated genes (Figure 2B). All DEGs are shown in the Supplementary Materials Table S2. To study the functions of the DEGs, we decreased the *p*-value cutoff to ≤0.01 and doubled the [log2 (FC)] > 2 value for the three groups. Within control vs. SARA, a total of 215 DEGs were obtained, of which 123 were upregulated and 92 were downregulated (Supplementary Materials Table S3). There were 43 DEGs, including 35 downregulated and 8 upregulated DEGs, in the control vs. ARA comparison. Likewise, for SARA vs. ARA, a total of 266 DEGs, with 90 up- and 176 downregulated DEGs, were reported (Table S2). Table 2 shows three groups of DEGs screened under [log2 (FC)] > 2, *p* < 0.01.

### 3.4. Functional Analysis of 215 DEGs Observed in Control vs. SARA

For control vs. SARA, a total of 215 DEGs were significantly enriched in GO terms ([log2 (FC)] > 2, *p* < 0.01), including 160 biological processes (BP), 1 cellular component (CC), and 25 molecular functions (MF) (*p* < 0.01). Among BP, major molecular functions were enriched, e.g., regulation of glucose transmembrane transport, regulation of leukocyte apoptotic process, negative regulation of the multicellular organismal process, regulation of signaling, regulation of MAPK cascade, and regulation of the metabolic process. The MFs found to be associated with DEGs included double-stranded DNA binding, sequence-specific DNA binding, regulatory region nucleic acid binding, and protein binding. The only CC associated with DEGs was the nucleus. A complete list of GO terms and genes involved is given in the Supplementary Materials Table S4.

**Figure 3:**
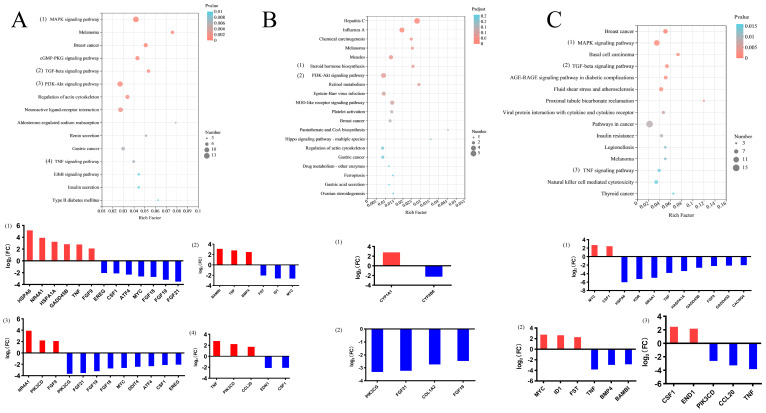
Kyoto Encyclopedia of Genes and Genomes (KEGG) analysis of the three groups. The top fifteen KEGG pathways significantly enriched in control vs. SARA (**A**), control vs. ARA (**B**), and SARA vs. ARA (**C**) and DEGs in the main KEGG pathways. The significance of the identified KEGG pathways was determined by *p* < 0.05. Red bars indicate upregulated genes and blue bars indicate downregulated genes.

Under the same conditions, a total of 43 pathways were significantly enriched in response to pH treatments (*p* < 0.05); inflammation was mainly related to the MAPK signaling pathway (e.g., HSPA6, NR4A1, HSPA1A, KDR, GADD45B, TNF, FGF9, GADD45G, CACNG4, CSF1, and MYC) and TNF signaling pathway (e.g., TNF, PIK3CD, CCL20, EDN1, and CSF1). The number of DEGs involved in these 43 KEGG pathways is presented in the Supplementary Materials Table S5. The top fifteen enriched KEGG pathways are shown in Figure 3A.

### 3.5. Functional Analysis of 43 DEGs Observed in the Control vs. ARA Groups

Forty-three genes were significantly differentially expressed in the control group compared with the ARA group ([log2 (FC)] > 2, *p* < 0.01). A total of eight BP and one MF (*p* < 0.01) were significantly enriched in the control vs. ARA comparison. Out of the eight BP, major gene portions were enriched in negative regulation of viral genome replication, negative regulation of viral life cycle, regulation of viral genome replication, regulation of ribonuclease activity, and immune effector process. The only MF was 2′-5′-oligoadenylate synthetase. A complete set of GO terms and DEGs involved are given in the Supplementary Materials Table S4.

A total of 14 pathways were significantly enriched in response to pH treatments (*p* < 0.05) (Supplementary Materials Table S5), including steroid hormone biosynthesis (e.g., CYP1A1 and CYP2B6) and the PI3K-Akt signaling pathway (e.g., PIK3CG, FGF19, COL1A2, and FGF21). Three commonly shared pathways were reported between the control vs. SARA and control vs. ARA comparisons. Furthermore, our findings revealed that most DEGs in these pathways were relevant to the PI3K-Akt signaling pathway. The top fifteen enriched KEGG pathways are shown in Figure 3B.

### 3.6. Functional Analysis of 266 DEGs Observed in SARA vs. ARA

Two hundred and sixty-six DEGs ([log2 (FC)] > 2, *p* < 0.01), a total of 79 BP, and 15 MF were detected in SARA vs. ARA (*p* < 0.01). One hundred seventy-six downregulated genes, e.g., HSPA5, BAIAP2, DNAJB1, and TXNIP, and 90 upregulated genes, e.g., WSB1, EIF5, and MYC, were significantly involved in all GO terms. Similarly, the GO terms for BP included structural constituents of negative regulation of nucleic acid-templated transcription, regulation of the biosynthetic process, regulation of the cellular process, and regulation of response to the stimulus. The MF-related GO terms consisted of protein folding chaperone, DNA-binding transcription factor activity, regulatory region nucleic acid binding, and DNA binding. The Supplementary Materials Table S4 provides a complete list of GO keywords and the DEGs implicated.

The KEGG pathway analysis results showed that 30 pathways were associated with the MAPK signaling pathway (e.g., TNF, HASPA1A, GADD45B, FGF9, and CACNG4), TNF signaling pathway (e.g., GADD45A, MYC, CSSF1, HSP6, KDR, NR4A1, TNF, BMP4, HASPA1A, GADD45B, FGF9, and CACNG), and TGF-bate signaling pathway (e.g., MYC, ID1, FST, TNF, BMP4, and BAMBI) in the SARA vs. ARA comparison (*p* < 0.05) (Supplementary Materials Table S5). The top fifteen enriched KEGG pathways are shown in Figure 3C.

### 3.7. Functional Analysis of 94 Shared DEGs in BRECs between “Control vs. SARA” and “Control vs. ARA”

A total of 1381 and 283 significantly ([log2 (FC)] > 1, *p* < 0.05) DEGs were documented in the two comparisons of “control vs. SARA” and “control vs. ARA”, respectively. Of these DEGs, 94 genes were commonly shared among the three comparisons. A total of 20 BP and 3 MF terms were significantly enriched (*p* < 0.05), and most were major molecular functions, e.g., immune effector processes, defense responses, regulation of transport, regulation of cell communication, response to organic cyclic compounds, and response to stress. Seven downregulated DEGs (e.g., PIK3CG, ATF4, PCK2, FIBIN, NRIP1, SLC7A11, IFIT1, DDIT4, and TMF1) and one upregulated DEG (e.g., CYP1A1) were enriched in BP and are associated with the response to organic cyclic compounds. The three major molecular functions related to DEGs consisted of receptor-ligand activity, signaling receptor activator activity, and receptor regulator activity. A complete set of GO terms and DEGs involved are provided (Supplementary Materials Table S6).

**Figure 4:**
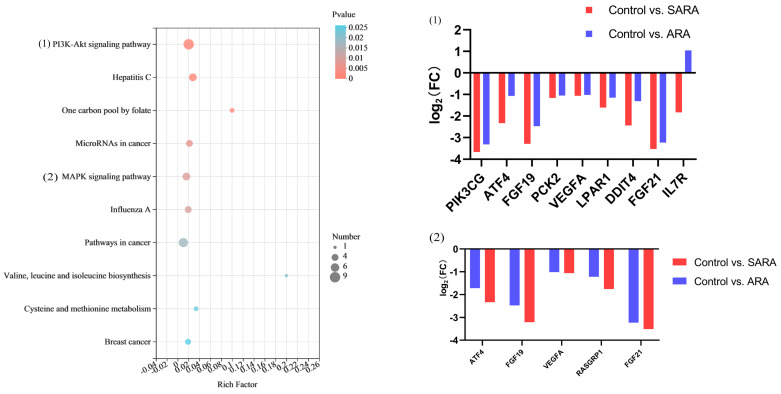
Significant KEGG pathways of 94 shared DEGs in BRECs between “control vs. SARA” and “control vs. ARA”. The significances of the identified KEGG pathways were determined by *p* < 0.05. Red bars indicate upregulated genes and blue bars indicate downregulated genes.

The results of KEGG pathway analysis showed that 18 pathways (*p* < 0.05) (Supplementary Materials Table S6), mainly related to PI3K-Akt signaling pathways (e.g., PIK3CG, ATF4, FGF19, PCK2, VEGFA, LPAR1, DDIT4, FGF21, and IL7R), and MAPK signaling pathways (e.g., ATF4, FGF19, VEGFA, RASGRP1, and FGF21), were significantly enriched; the top ten KEGG pathways are shown in Figure 4.

Quantitative reverse transcription-polymerase chain reaction (RT-qPCR) analyses of six random genes were carried out on the same samples to corroborate the expression changes of genes found in the RNA-Seq data. The results demonstrated that all of these genes’ expression patterns matched those discovered by RNA-Seq. The dependability of our RNA-Seq data was indicated by the consistency between the RT-qPCR and RNA-Seq results (Supplementary Materials Figure S1).

### 3.8. PPI Network Analysis

To gain more understanding of the interaction between the DEG-regulated pathways under low-pH stress, an interaction network for the proteins corresponding to the DEGs was constructed using STRING analysis with medium confidence (0.4) for all comparisons (e.g., control vs. SARA, control vs. ARA and SARA vs. ARA). The PPI interaction network analysis revealed that the majority of the proteins in SARA vs. ARA were highly interconnected when compared to control vs. SARA and SARA vs. ARA. (Supplementary Materials Figure S2A–C). The PPI in response to SARA treatment showed that TNF, MYC, FST, BMP4, PIK3CG, FGF9, FGF19, HSP1A1, and PIK3CD were distributed in the central parts. In the ARA-treated GC culture group, PPI network analysis revealed that CXCL8, ATF3, CYP1B1, TLR2, IFIT1, IFIT3, IFIT2, RNASEL, and OAS1Y occupied the central position. TNF, CCL20, BMO4, HSP1A1, CXCL8, END1, EGR1, and AFT3 were also strongly connected in the network center.

### 3.9. Identification of Differential Metabolites

**Figure 5:**
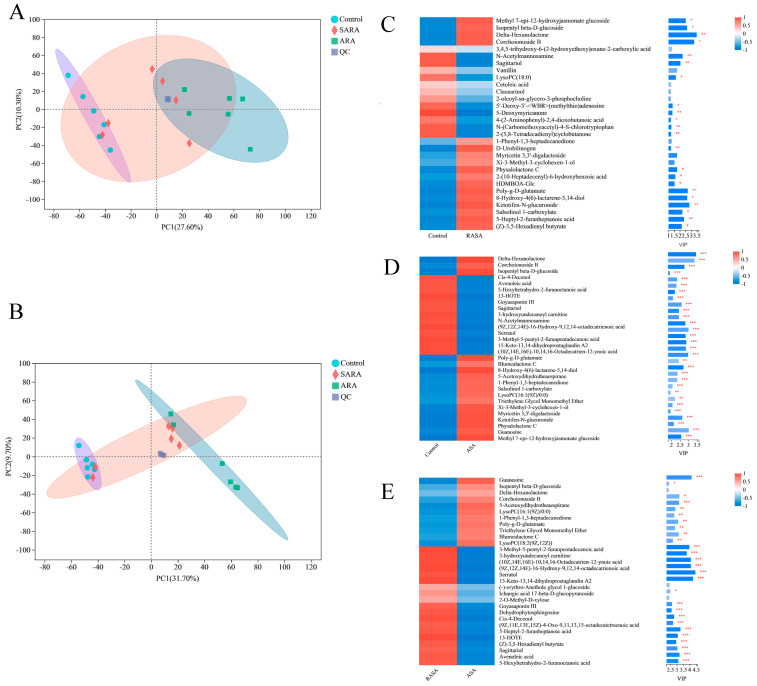
Principal component analysis (PCA) score plots of metabolomic data (**A**,**B**) and variable importance of the projection (VIP) scores of spermatozoa metabolites (**C**–**E**). (**A**) Positive ionization modes. (**B**) Negative ionization modes. Green = control group; blue = SARA; yellow = ARA; orange = QC; QC = quality control samples. (**C**) Control vs. SARA. (**D**) Control vs. ARA. (**E**) SARA vs. ARA. Each column represents one sample and the sample names are shown below each column. Each line represents one metabolite, and the colors represent the relative expression amounts of the metabolites in the group of samples. See the gradient color block for the corresponding relationship between the color gradient and value size. A metabolite VIP bar chart is shown on the right. Bar lengths indicate the contribution values of the metabolites to the difference between the two groups. By default, VIP > 1. The greater the value is, the greater the difference between the two groups. The bar color intensity denotes the statistical significance of the difference in metabolites between the two groups. * stands for *p* < 0.05, ** stands for *p* < 0.01, and *** stands for *p* < 0.001.

LC-MS was used to perform metabolite profiling of BRECs and 414/350 metabolites were identified in ESI ± mode. Principal component analysis (PCA) of the metabolome data showed that the two treatment groups were separated from the control group in ESI± mode and all of the samples in the score plots were within the 95% Hotelling T-squared ellipse, indicating that few outliers were present (Figure 5A,B).

Metabolites were screened as significantly distinct metabolites if their variable importance in the projection (VIP) values were more than 1 and *p* < 0.05. By combining the ESI ± LC-MS/MS data, 153 significantly different metabolites were found in the control vs. SARA comparison, 195 were found in the control vs. ARA comparison, and 124 were found in the SARA vs. ARA comparison. A complete set of metabolites is shown in the Supplementary Materials Table S7. Figure 5 shows the VIP value analysis of three different metabolites of the top thirty. KEGG enrichment analysis was carried out for different metabolites in each group, and the results are shown in Table 3. 

### 3.10. Integrated Analysis of the Transcriptome and Metabolome

**Figure 6:**
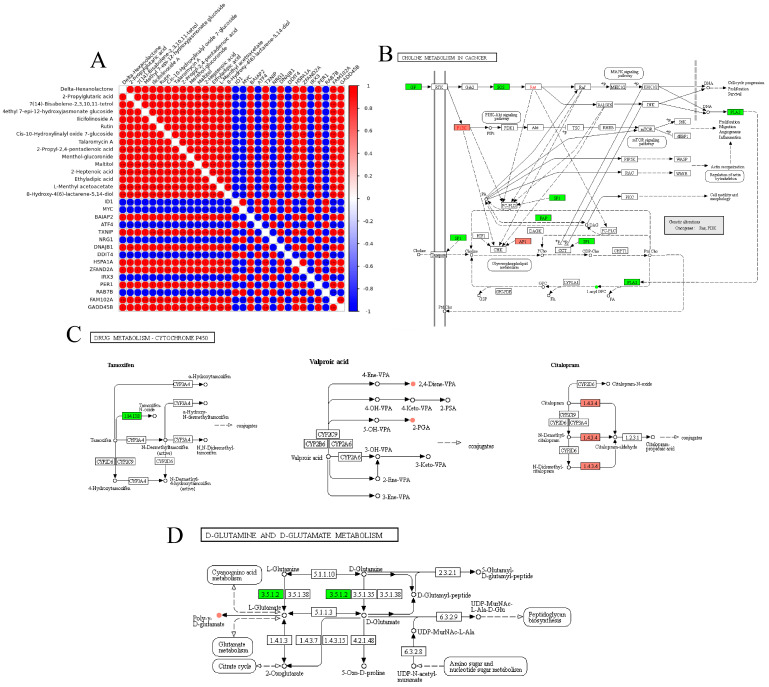
Integrated analysis of the expressions of differentially expressed genes (DEGs) and metabolites in control vs. SARA. (**A**) Pearson correlation analysis of the expressions of differentially expressed genes (DEGs) and metabolites (TOP15). Red indicates a positive correlation, blue indicates a negative correlation, and deeper colors indicate stronger correlations. (**B**) Part of choline metabolism in cancer. (**C**) Drug metabolism-cytochrome P450 pathway. (**D**) D-Glutamine and D-glutamate metabolism pathways. The round nodes are metabolites and the square nodes are the enzymes corresponding to the transcripts. The expression fold changes of metabolites from low to high are represented by blue to yellow colors, and the expression fold changes of transcripts from low to high are represented by green to red colors.

In control vs. SARA, comparing the transcriptomic and metabolomic data showed that highly positive correlations were obtained for most of the differential metabolites. ID1, MYC, ATF4, NRG1, DDIT4, IRX3, and RAB7B were negatively correlated with differential metabolites. There were also high correlations among DEGs (Figure 6A). Maps of all DEGs and differential metabolites into KEGG that were significant enrichment pathways between control vs. SARA, including D-Glutamine and D-glutamate metabolism, Choline metabolism in cancer and Drug metabolism-cytochrome P450, are shown in Figure 6B–D.

Similarly, in the control vs. ARA comparison, highly positive correlations were observed among most differential metabolites and DEGs of CYP1A1 and FAM89B; the only exception was (9Z,12Z,14E)-16-hydroxy-9,12,14-octadecatrienoic acid, which was negatively correlated with all other differential metabolites. Instead, (9Z,12Z,14E)-16-hydroxy-9,12,14-octadecatrienoic acid was negatively correlated with CYP1A1 and FAM89B (Supplementary Materials Figure S3A). All DEGs and differential metabolites were mapped to KEGG and purine metabolism, the PPAR signaling pathway, and alpha-linolenic acid metabolism were significantly enriched (Supplementary Materials Figure S3B–D).

## 4. Discussion

The overall goal of this study was to determine how the pH level in the rumen affects mRNA expression and metabolite changes of these cytokines in vitro. The cause of rumen acidosis is a decrease in rumen pH caused by dietary changes, which further leads to release of lipopolysaccharide (LPS) by rumen microorganisms [36]. LPS can also shift from the rumen to the peripheral circulation and activate inflammatory responses [11,37]. However, LPS is not the primary factor inducing acidosis, and the pH change occurred earlier and was the reason for LPS release. 

### 4.1. Effect of pH Stresses on BREC Transcription and Metabolism

In the present study, the preliminary finding is that low pH reduces the viability of BRECs in the two treatment groups and causes apoptosis in ARA groups. This could be explained by endoplasmic reticulum stress and altered mitochondrial membrane potential, which induces apoptotic signaling [38]. Of the several hundred DEGs induced or repressed during pH stress in vitro, the transcription analysis showed that they were primarily associated with inflammation, cell regulation, and apoptosis, including elevated protein abundance of acidified NF-kappa B p65, IL-6, and tumor necrosis factor (TNF), which were consistent with previous studies [39]. It is worth noting that the expressions of DEGs connected with inflammation were higher at SARA pH levels than at the pH of the ARA group; this may be due to significant apoptosis in the ARA group of cells. 

In the SARA group, the KEGG analysis showed that pathways associated with inflammation included the MAPK signaling pathway and TNF signaling pathway. Our results showed an increased significance (*p* < 0.05) of inducible TNF in SARA groups at the mRNA level [17]. Likewise, several inflammation-related genes, such as JUN, PKA, and MAPK3K, were also significantly upregulated; NF-κB was downregulated under pH 5.5 stress, which signals the involvement of the inflammatory signaling pathway [40,41,42,43]. TNF is a proinflammatory cytokine. The goal of inflammation is to wipe out the irritating agent and stimulate tissue regeneration. An inadequate inflammatory reaction can lead to tissue damage and in severe cases, even to organ failure and death [44]. TNF receptors (TNFRs) are a subset of the TNF receptor superfamily (TNFRSF), which mostly consists of TNFR1 and TNFR2. Responses to TNFR1 stimulation may be inflammatory or apoptotic, while TNFR2 is particularly important for cell activation, migration, and proliferation [45]. TNF can activate NF-κB via the IκB kinase (IKK: an inhibitory protein of NF-κB kinase) complex [46]. Similarly, TNF can activate JNK via either caspase-dependent or caspase-independent pathways [47]. An essential branch of the MAPK pathway is the JNK signal transduction pathway. The JNK signal pathway is crucial for numerous physiological and pathological processes, including cell stress, apoptosis, reproduction, and the cell cycle [48]. In particular, JNK regulates the production of TNF and can regulate the inflammatory response through the expression of e-selectin [49,50]. This shows that under in vitro conditions, BRECs can regulate inflammation through the TNF/MAPK pathway at pH 5.5. In addition, we noted that the PI3K gene was upregulated and TLR4 was downregulated in the PI3K-AKT signaling pathway. The PI3K-PKB signaling pathway can promote cell survival; activated PKB can directly phosphorylate precursor apoptotic proteins and can, in the short term, prevent activation of the apoptotic pathway, leading to cell death [51]. CCL20 was also upregulated in the SARA group compared with the control and ARA groups. CCL20, as a chemokine, can participate in the NF-kappa B and Akt signaling pathways to regulate cellular inflammation [52,53]. However, it was significantly downregulated in the ARA group compared to the control group. This may be the result of a general breakdown of cellular homeostasis in the ARA group. Many results show that BRECs regulate their inflammatory response through other pathways in the pH 5.5 environment to maintain their survival and physiological functions. 

The PI3K-Akt signaling pathway and TNF signaling pathway were also enriched in the ARA group. Our results showed that the ARA group significantly induced apoptosis. In combination with the transcriptional results, data on the induced expression of genes at pH = 4.5 suggest that the cellular mechanism may not protect BRECs. To study the differences in apoptosis between the SARA and ARA groups, we examined the expressions of apoptotic genes. It is worth noting that the FAS expression of the ARA group was higher than that of the SARA group. FAS is a transmembrane protein and binding FASL can initiate transduction of the apoptosis signal and cause apoptosis [54]. Studies have shown that upregulation of apoptotic genes could lead to disruption of the potential of the mitochondrial transmembrane, resulting in cytochrome c release and leading to apoptosis induction [55]. Acidosis in the extracellular environment can induce cell death through a pathogenic mechanism mediated by the opening of ROS and the mitochondrial permeability transition pore (mPTP) [56]. Similar to the results of our study, an abundance of proapoptotic response-related proteins (e.g., cytochrome c, BAX, and caspase-3) was upregulated in the rumen in a low-pH environment [39]. Data on the induced expression of apoptotic genes at pH = 4.5 suggest that the cellular mechanism may not protect BRECs against pH-induced apoptosis, while the rate of apoptosis decreased at pH = 5.5 due to upregulation of PI3K; the HSP90 mRNA levels likely helped BRECs activate self-protection mechanisms. Therefore, we suggest that BRECs mediate inflammation through the TNF/MAPK pathway and resist external stimulation in a low-pH environment. Our results agree with a previous report in which the TNF/MAPK pathway at low pH could play a crucial role in inflammation [57]. 

In addition, the results of 94 shared DEGs observed both in the “control vs. SARA” and “control vs. ARA” groups showed that cytochrome P450 was also affected by the low-pH treatment. In our study, CYP1A1 was significantly upregulated in the control vs. ARA group and tended to increase in the control vs. SARA group, while CYP26A1 was downregulated in both groups. CYP1A1 is a member of the cytochrome P450 subfamily. Previously, it was reported from clinical research that the P450 subfamily was involved in tumor deterioration and related diseases, and it was also found that the P450 enzyme has certain activity changes in the pathogenesis of many inflammatory diseases [58]. CYP1A1 was downregulated in hepatocytes in an LPS model in vitro [59]. Based on this confirmation, we postulate that regulation of CYP11A1 may indeed participate in the inflammatory response. The transcriptional expression profile of the RNA-Seq analysis in the current study was verified by the RT-qPCR analysis.

The metabolome data further showed that the control group had significant metabolic differences compared with the SARA and ARA groups. The drug metabolism–cytochrome P450 pathway was enriched simultaneously. Although there are few studies about drug metabolism involving the cytochrome P450 pathway in BRECs, studies on the CYP2 gene upstream of its pathway show that it can participate in the I3K/Akt/mTOR signaling pathway [60], which is consistent with the transcriptome results. Hence, we speculate that the functional involvement of the P450 family may be one of the mechanisms of inflammation induced by low pH, although the detailed mechanism needs to be further studied. In addition, we found that the differential metabolites in the two groups were mainly concentrated in lipid compounds. Fatty acids can regulate lipid metabolism and immune response in the rumen epithelium [61]. The aforementioned findings thus support the crucial function of the rumen epithelium’s metabolic adaption to low pH.

### 4.2. Integrated Analysis of the Transcriptome and Metabolome

The mechanisms underlying metabolite changes in rumen acidosis and the associated genetic mechanisms provide insight into the association between genes and the regulation of the expression of important metabolites [62,63]. In this study, further correlation analysis revealed that several metabolic pathways were specifically enriched in the SARA and ARA groups, illustrating the changes in metabolism associated with the pH stress response of BRECs. In control vs. SARA, FMO3 (flavin containing dimethylaniline monooxygenase 3) was significantly downregulated and MAOA (monoamine oxidase A) was upregulated in drug metabolism−cytochrome P450. FMO3 activity is similar to that of the CYP family and is an important process for the oxidation of metabolites in many cases [64]. For choline metabolism in the cancer pathway, SOS Ras/Rho guanine nucleotide exchange Factor 2 (SOS2) was significantly upregulated, and receptor-induced membrane recruitment of the cytoplasmic protein SOS led to Ras/MAPK cascade activation [65], which may be one of the ways that BRECs activate the MAPK pathway in the face of low-pH stress. In addition, pH = 5.5 also causes changes in D-glutamate metabolism in rumen epithelial cells. Glutamate is essential for cellular physiology. The catabolism and anabolism of other amino acids are both related to glutamate metabolism [66], and the TCA cycle metabolite α-ketoglutarate is produced when glutamate is deaminated. This is an essential anaplerotic pathway [67]. 

In the control vs. ARA comparison, the correlation analysis results showed that CYP1A1 was positively correlated with most differential metabolites and negatively correlated with differential genes, which suggests that there is a regulatory relationship among genes and metabolites. It has been reported that downregulation of CYP1A1 mRNA can alleviate the inflammatory damage of epithelial cells, accompanied by the NF-κB signaling pathway activation [68], which is consistent with the results of this experimental study. However, its specific regulatory mechanism needs to be further studied. DEGs and differential metabolites were significantly enriched in lipid and carbohydrate metabolic pathways in the control vs. ARA comparison. In particular, pH 4.5 caused significant changes in lipid metabolism and alpha-linolenic acid (ALA) was significantly lower than that of the control group. It is worth noting that FADS mRNA decreased significantly, and was enriched in alpha-linolenic acid metabolism and the PPAR signaling pathway. FADS2 is a key enzyme in the polyunsaturated fatty acid (PUFA) metabolism of alpha-linolenic acid (ALA) into eicosapentaenoic acid (EPA) [69]. It has been reported that FADS2 is also significantly correlated with inflammation, which needs further research [70].

## 5. Conclusions

In the present study, we demonstrated a method to characterize the cellular, transcriptomic, and metabolomic adaptations of BRECs to different pH stress intensities (pH = 5.5, 4.5) in vitro. The study results suggested that the pH = 4.5 treatment caused comparatively more apoptosis of BRECs, and both pH treatments (pH = 5.5, 4.5) changed the transcriptional regulation of BRECs. It was suggested that HASPA1A, TNF, and PIK3CG regulate the inflammatory response of BRECs through the MAPK/TNF signaling pathway under pH stress. Meanwhile, differential metabolite analysis showed that 2-propyl-2,4-pentadienoic acid and poly-g-D-glutamate were involved in the changing metabolic process. In general, the change in pH caused an inflammatory reaction of rumen epithelial cells, and the carbohydrate metabolism and amino acid metabolism also changed. The model of the BREC response to pH stress described in the current study provides a theoretical strategy to understand the inflammatory mechanisms of low-pH stress on BRECs when experiencing rumen acidosis.

## Figures and Tables

**Table 1:** Effect of pH treatment on BREC apoptosis (%).

Groups	Normal Cells	Necrotic Cell	Early Apoptotic Cells	Late Apoptotic Cells	Apoptosis Index
Control	72.3 ± 3.0 ^ab^	16.6 ± 2.6 ^ab^	2.1 ± 0.025 ^ab^	9.0 ± 0.4 ^a^	11.1 ± 0.4 ^a^
SARA	76.8 ± 1.8 ^a^	12.5 ± 1.8 ^ab^	2.4 ± 0.7 ^ab^	8.3 ± 0.5 ^a^	10.7 ± 0.7 ^a^
ARA	64.3 ± 4.2 ^b^	17.3 ± 2.7 ^ab^	3.2 ± 0.5 ^ab^	15.2 ± 1.3 ^b^	18.4 ± 1.5 ^b^

Note: Different letters ^a, b^ in the same columns differ significantly (*p* < 0.05) by Duncan’s test. Data are shown as the mean ± SEM.

**Table 2:** Number of DEGs discovered in three comparisons of BRECs after pH treatment.

Groups	Total Genes	Upregulated	Downregulated	Criteria
Control vs. SARA	215	123	92	[log2 (FC)] > 2, *p* < 0.01
Control vs. ARA	283	56	227	[log2 (FC)] > 2, *p* < 0.01
SARA vs. ARA	266	90	176	[log2 (FC)] > 2, *p* < 0.01

**Table 3:** Metabolic pathways of differential metabolites.

	Pathway Name	KEGG Map ID	*p* Value
Control vs. SARA	Drug metabolism—cytochrome P450	map00982	0.0112
Control vs. ARA	Asthma	map05310	0.0366
	PPAR signaling pathway	map03320	0.0366
	Autophagy—animal	map04140	0.0293
	Autophagy—other	map04136	0.0221
	Glycosylphosphatidylinositol (GPI)-anchor biosynth	map00563	0.0221
	alpha-Linolenic acid metabolism	map00592	0.038
	Bile secretion	map04976	0.0347
	Purine metabolism	map00230	0.0295
	Drug metabolism—cytochrome P450	map00982	0.0225
SARA vs. ARA	Asthma	map05310	0.0285
	PPAR signaling pathway	map03320	0.0285
	alpha-Linolenic acid metabolism	map00592	0.0237
	Phenylalanine metabolism	map00360	0.0076
	Bile secretion	map04976	0.0177

## Data Availability

The data used in this study are available from the corresponding author on request.

## Supplementary Materials

The following supporting information can be downloaded at: https://www.mdpi.com/article/10.3390/ani12192621/s1, Figure S1: Validation of RNA-Seq results by RT-qPCR; Figure S2: Protein-protein interaction (PPI) networks of DEGs significantly enriched pathways associated with BRECs function at different pH; Figure S3: Integrated analysis of the expressions of differentially expressed genes (DEGs) and metabolites in control vs. ARA; Table S1: Primers used in quantitative real-time PCR; Table S2: The significant DEGs of control vs. SARA, control vs. ARA, and SARA vs. ARA ([log2 (FC)] > 1, *p* < 0.05); Table S3: The significant DEGs of control vs. SARA, control vs. ARA, and SARA vs. ARA ([log2 (FC)] > 2, *p* < 0.01); Table S4: The significant GO terms for control vs. SARA, control vs. ARA, and SARA vs. ARA (*p* < 0.01); Table S5: The significant KEGG pathways of control vs. SARA, control vs. ARA, and SARA vs. ARA (*p* < 0.05); Table S6: The GO terms and KEGG analysis of 94 shared DEGs in BRECs between “control vs. SARA” and “control vs. ARA”; Table S7: The significant different metabolites of control vs. SARA, control vs. ARA, and SARA vs. ARA ([log2 (FC)] > 2, *p* < 0.05).
