# Supplementary material for: In Vitro Gene Expression Responses of Bovine Rumen Epithelial Cells to Different pH Stresses
Source: Animals (Basel). 2022 Sep 29;12(19):2621. doi: 10.3390/ani12192621 (PMC9559271; doi:10.3390/ani12192621)
Supplement: Supplementary file 1 [file animals-12-02621-s001.zip › Supplementary Materials Figure S1 S2 S3.pdf]

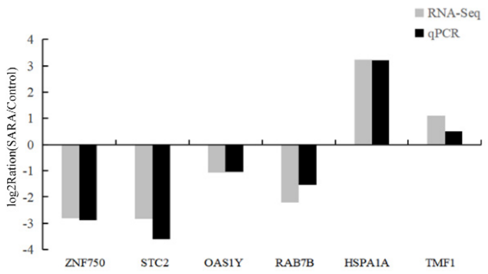

Figure S1. Validation of RNA-Seq results by RT-qPCR. Comparison of RT-qPCR findings to RNA-Seq results. Validation of differentially expressed genes by RT-qPCR. Relative quantification of 6 representative genes was performed. RT-qPCR values were determined from the ΔΔ Ct for the target genes.

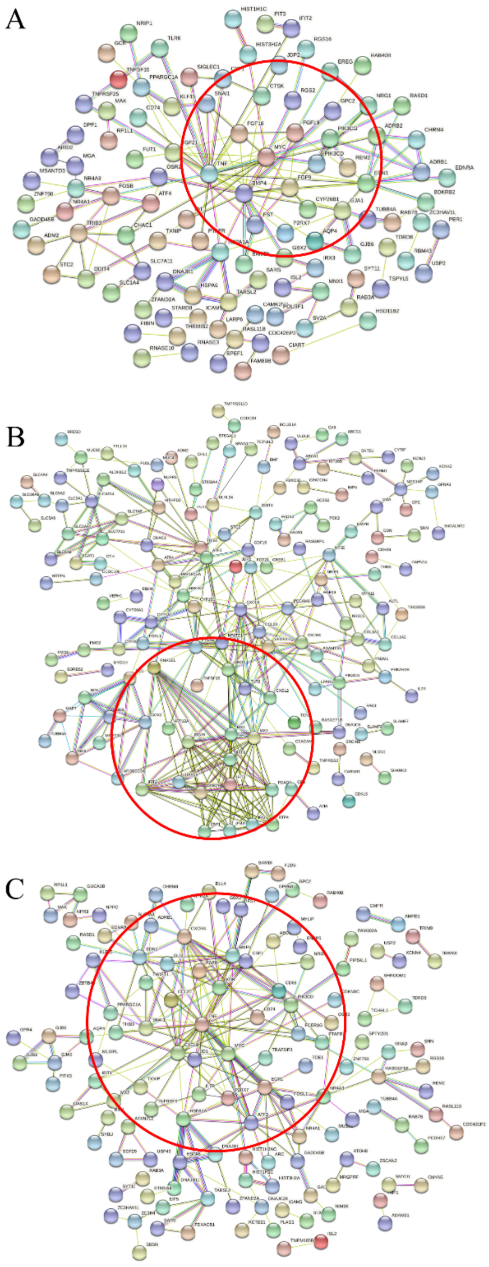

Figure S2. Protein-protein interaction (PPI) networks of DEGs significantly enriched pathways associated with BRECs function at different pH. Protein-protein interaction (PPI) networks in the comparison of “Control vs. SARA” (**A**) “Control vs. ARA” (**B**) and “SARA vs. ARA” (**C**). Various color lines represent seven types of evidence used in predicting associations. Red line: fusion evidence; blue line: co-occurrence evidence; yellow line: text mining evidence; green line: neighborhood evidence; purple line: experimental evidence; light blue line: database evidence; and the black line: co-expression evidence.

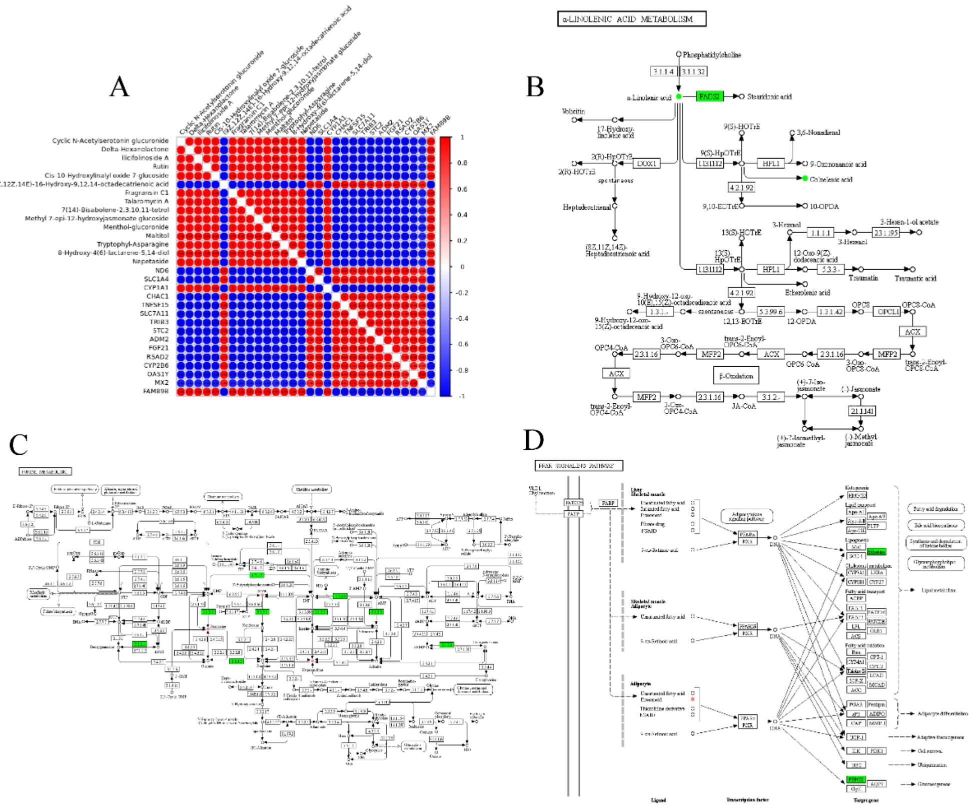

Figure S3. Integrated analysis of the expression of differentially expressed genes (DEGs) and metabolites in Control vs. ARA. (**A**) Pearson correlation analysis of the expression of differentially expressed genes (DEGs) and metabolites (TOP15). Red indicates a positive correlation, and blue indicates a negative correlation, a deeper color indicates stronger correlations. (**B**) alpha-Linolenic acid metabolism. (**C**) Purine metabolism. (**D**) PPAR signaling pathway. The round nodes are metabolites, and the square nodes are the enzymes corresponding to the transcripts. The expression fold change of metabolites from low to high is represented by blue to yellow, and the exprsession fold change of transcripts from low to high is represented by green to red.
