# Supplementary material for: In Vitro Gene Expression Responses of Bovine Rumen Epithelial Cells to Different pH Stresses
Source: Animals (Basel). 2022 Sep 29;12(19):2621. doi: 10.3390/ani12192621 (PMC9559271; doi:10.3390/ani12192621)
Supplement: Supplementary file 1 [file animals-12-02621-s001.zip › Supplementary Materials Table S1.pdf]

Table S1 Primers used in quantitative real-time PCR

Gene	Primer	Forward primer (5'-3')	Length（bp）	GeneBank ID
ZNF750	F	GGTATCTCCGAAAAGCCTCG	131	NM_001081533.1
	R	CCTCTGGTTTCGTGGACAGG		
STC2	F	CCTTGCAGAACACAGCGGAA	104	NM_001192745.3
	R	GCCCCGAATCTCACAAGAGT		
OAS1Y	F	CAAATAGCTGGGAGCGGCTT	116	NM_001040606.1
	R	ACTGTGTTCTTGGGGCGACA		
RAB7B	F	GGCTCTGATGGTTGTGTCCT	168	XM_024976353.1
	R	GCTTCGATACTGCCGGTCTT		
HSPA1A	F	GTGCAGGAGGCGGAAAAGTA	100	NM_203322.3
	R	GGAAATCACCTCCTGGCACT		
TMF1	F	AGAAAAGGAGCGGAAACCGT	199	NM_001206260.1
	R	TGATCCCGCTCCCATCCTTA		
GAPDH	F	CGATGCCCCCATGTTTGTGA	149	NM_001034034.2
	R	CATGAGCCCCTCCACAATG		

Notes: F and R refer to the forward and reverse primers, respectively.
